# Impact of tissue processing on microbiological colonization in the context of placentophagy

**DOI:** 10.1038/s41598-022-09243-4

**Published:** 2022-03-29

**Authors:** Sophia K. Johnson, Jana Pastuschek, Daniel C. Benyshek, Yvonne Heimann, Anne Möller, Jürgen Rödel, Jacob White, Janine Zöllkau, Tanja Groten

**Affiliations:** 1 Department of Obstetrics, Placenta Lab, Jena University Hospital, Am Klinikum 1, 07747 Jena, Germany; 2 Department of Anthropology, University of Nevada, Las Vegas, USA; 3 School of Medicine, University of Nevada, Las Vegas, USA; 4 Institute of Medical Microbiology, Jena University Hospital, Jena, Germany

**Keywords:** Medical research, Preclinical research

## Abstract

A mother’s postpartum ingestion of raw or processed placental tissue—referred to as human maternal placentophagy—is an emerging health trend observed in industrialized nations. Placenta is commonly consumed as small pieces of raw tissue, or as raw or steamed dehydrated pulverized and encapsulated tissue. To investigate the potential neonatal health risks of this behavior, the present study focused on microbial colonization of processed placenta preparations with potentially pathogenic bacteria *Streptococcus agalactiae* (Group-B-Streptococci; GBS) and *Escherichia coli* (*E. coli*). In the clinical approach placentas from 24 mothers were analyzed. Two placentas, from 13 mothers with confirmed positive maternal GBS status, showed GBS-growth on their surface (2/13; 15.4%) independent from delivery mode or antibiotic treatment. All processed samples (n = 24) were free from GBS. In the experimental approach, a standardized inoculation protocol was introduced to resemble ascending vaginal and hematogenous colonization. Six placentas from elective term C-sections of GBS negative mothers were collected and artificially inoculated with highly concentrated suspensions of GBS and *E. coli*. Heat processing significantly reduced the number of colony forming units (CFU) for GBS and *E. coli*. Our results suggest placentophagy of processed tissue is an unlikely source of clinical infection.

## Introduction

Human maternal placentophagy, the mother’s consumption of their placenta postpartum, is an emerging alternative health-seeking trend in post-industrial nations^1–3^. While there is evidence of processed human placenta being used as a medical remedy for centuries in Chinese Medicine^4^, it is indicated for a host of ailments in this context, and prescribed to both women and men. The use of dehydrated placenta tissue in German traditional midwifery practice^5^ as a remedy during “difficult birth” is described in a pharmacopoeia from 1741^6^. In contrast, the first well documented instances of placenta consumption by the peripartum mother (consistent with the ubiquitous mammalian maternal behavior^7^) appear as a rare, alternative maternal health practice in the 1970s among women in the US home-birthing community. In the US, almost one third of women from outpatient deliveries consume their afterbirth^3^, although scientific evidence about health risks and benefits of placentophagy is limited. Human placentophagy studies have focused on hormones^8,9^, microorganisms^9^ and trace elements^9,10^, and potential effects placentophagy may have on fatigue^11^, iron-status^12^ and mental health status^11^ of postpartum mothers. These studies include self-reported survey data of placentophagic participants^2^, in addition to laboratory placental tissue analyses^8,10^ and a phase 1 randomized clinical trial^11–13^. In light of the limited number of studies assessing the safety of placentophagy, some practitioners advocate discouraging the practice^14^. The microbiologic aspects of placentophagy, however, remain largely unexplored.

Neonatal GBS infection may subsequently lead to either early- or late-onset GBS disease. Onset of symptoms within the first six days of life defines early onset GBS disease (EOD); a vertical transmission from colonized mothers sub partu directly to the neonate is postulated. Late onset GBS disease (LOD) is characterized by onset from seven through 89 days of life. A transmission route for LOD may result from an exogenous source such as breast milk, or by fecal–oral transmission, or even may result from established colonization^15^.

Colonization is the presence of GBS on a body site like the gastrointestinal or genitourinary tract without causing disease. Approximately 18% of pregnant women are asymptomatic colonized recto-vaginal with GBS worldwide^16^. Infection is the invasion of the host’s tissues with pathogen microbes, characterized by clinical symptoms and laboratory evidence. Host-specific factors might play a role in determining an individual’s susceptibility to invasive disease^17^. However, as colonization is a risk factor for an ascending infection from the vagina to the fetus, the CDC recommended universal, culture-based screenings in 2002. The German guideline for prevention of GBS disease is consistent with the CDC recommendations from 2016^18^. A rectovaginal swab is performed between week 35 + 0 and week 37 + 0 of pregnancy by midwives or obstetricians according to the screening recommendations for prepartum care^18^. The microbial analysis is similar to the method described in “Methods” section.

In the case of a positive recto-vaginal GBS culture in the third trimester of pregnancy, antibiotic treatment with e.g. ampicillin should be administered especially after premature rupture of membranes (PROM).

A case report published by the US Centers for Disease Control and Prevention (CDC) in 2017^19^ discussed maternal placentophagy as a risk factor for neonatal GBS infection acquired from encapsulated pulverized placenta tissue. In this case, a term born infant from uncomplicated pregnancy with the mother tested negative for GBS in week 37 suffered from EOD on day one postnatal, with GBS positive blood cultures. After treatment with ampicillin, recovery, and discharge from hospital, the newborn was readmitted 16 days postnatal due to general irritability and fever. LOD was diagnosed after blood cultures were again positive for GBS. Genotyping of GBS isolated from the placenta capsules consumed by the mother revealed the same strain as in the neonatal blood sample, while breast milk was free from GBS. The case study authors concluded that consuming the placenta postpartum likely caused high maternal GBS colonization that led to LOD in the newborn.

It should be noted however, that a large medical records observational study in the US comparing placenta consumers (n = 7162) versus non-consumers (n = 10.951) found no significant difference in neonatal hospitalizations, neonatal intensive care admissions, or neonatal death at 6 weeks postpartum between the two groups^3^.

*Streptococcus agalactiae* is the species designation for the Lancefield Group B Streptococci (GBS), a commensal bacteria of the digestive and genitourinary tract of humans^20^. GBS are characterized by the surface capsular polysaccharide into 10 serotypes (Ia, Ib, and II–IX). The distribution of serotypes varies in distinct regions worldwide and can change over time^21^. Several virulence factors like the polysaccharide layer, hair-like pili, and the toxin beta-hemolysin help GBS to attach to the hosts cells and invade its immune system^22^. A predominant clone belongs to serotype III, subtype 2 (III-2), sequence type 17, which is less genetically diverse than strains from the other serotypes^23^. This type is described as a hypervirulent GBS clone that is strongly associated with meningitis and late onset neonatal infections^23,24^ and which was also identified in the CDC case report cited earlier^19^.

Besides GBS the bacterial species *Escherichia coli* (*E. coli*) serves as the main source of neonatal infection. *E. coli* is a gram-negative commensal of the intestinal microflora of healthy individuals with a wide geno- and phenotypic diversity. Depending on its serotype it can be facultative pathogen and the cause of extraintestinal or intraintestinal infections^25^.

*E. coli* and GBS are the most frequent pathogens responsible for neonatal infection leading to admission to neonatal intensive care unit. Ambivalent data exists on the growing incidence of *E. coli*- sepsis and its predominance in early onset neonatal sepsis (EONS) of very low birth weight infants^26–28^. As *E. coli* is a common species found ubiquitous in the rectovaginal flora, a screening for this bacteria is not routinously performed in pregnancy as known for the GBS screening.

The human digestive system acts as a barrier against ingested microbes. The stomach is well known for its acidic milieu that, while not sterile, has an estimated pH of 2.0^29^, thereby lowering the quantity of microorganisms and decreasing the risk of infections by pathogens^30,31^. GBS is sensitive to acidic environment and heat preparation^32^, although little data exists on GBS and its properties on placental tissue^33^. Most women who engage in placentophagy consume placental tissue raw-dehydrated, ground and filled into capsules (48.4%) and one third use capsules that contain dehydrated ground tissue which was steamed before; also the ingestion of raw tissue is documented^3^.

The present study aims to evaluate the microbial colonization of placental tissue and to answer the question of whether or not GBS and *E. coli* can survive different placenta preparation processes (raw; steamed; raw-dehydrated; and steamed-dehydrated). Processing raw tissue includes rinsing the placenta under cold running water and homogenizing the tissue; dehydration is low heat preparation at 55 °C for a minimum of 8 h and steamed-dehydrated tissue is heated over water vapor for minimum 10 min until the core temperature is 70 °C or above (compare Fig. 3 and “Methods” section^3^. In a clinical approach placenta samples from mothers with different GBS-status were processed as described and microbial colonization was analyzed. In order to investigate the impact of the two hypothesized routes of infection on microbial transmission we performed an experimental approach*. S*tandardized protocols for contamination according to the vaginal as well as for the hematogenous route were introduced. Placenta samples were artificially inoculated with GBS and *E. coli* before processing as described.

## Results

### Participant characteristics clinical approach

24 placentas were analyzed, 16 placentas were from vaginal deliveries, eight were from C-sections. 17 placentas were from mothers being documented as GBS positive by maternity records, seven placentas were obtained from unscreened mothers with unknown GBS-status. Baseline data and results of GBS diagnostics are shown in Table 1 grouped according to GBS-status (maternity record) and delivery mode.

**Table 1:** Baseline data and results according to GBS-status (maternity record) and delivery mode and antibiotic treatment.

	Delivery mode	Antibiotic treatment	Group*	Processed	GBS** confirmed vaginal/rectal	GBS** on placenta	GBS** in processed samples
GBS positive by maternity record	Vaginal delivery	No	I	3	1	–	–
		Yes	II	10	8	1	–
	C-Section	No	III	4	3	1	–
Unscreened for GBS	Vaginal delivery	No	IVa	3	–	–	–
	C-Section	No	IVb	4	1	–	–
Sum				24	13	2	0

*Group I: GBS positive, vaginal delivery, no antibiotic treatment, (II) GBS positive, vaginal delivery, antibiotic treatment, (III) GBS positive, C-section, no antibiotic treatment, (IVa) unscreened for GBS, vaginal delivery, no antibiotic treatment, (IVb) unscreened for GBS, C-section, no antibiotic treatment.

**GBS confirmation was done by maternal vaginal/rectal swabs at time of birth. GBS were analyzed in all samples by culture and isothermal loop-mediated amplification (LAMP).

From 17 mothers with positive GBS status according to their maternity record, standard routine cultivation and/or GBS-specific PCR maternal GBS-colonization was confirmed in 12 cases (70.6%) by vaginal and/or rectal swab at the time of delivery. One rectal swab from an unscreened mother was subsequently GBS positive. Two placentas out of 13 with confirmed maternal GBS-colonization showed GBS-growth on their surface (2/13; 15.4%): one placenta was born vaginally with antibiotic treatment, the other placenta via C-section without antibiotic treatment.

Participant and neonatal characteristics are described in Table 2. One case (P18; Supplement 1) was excluded from Group IVb because of preterm birth with following characteristics: C-section, mother unscreened for GBS, no GBS detection vaginal/rectal, preterm infant (34 + 3 wks), Gravida/Para status II/I, weight 1835 g, length 42 cm, head circumference 29 cm, APGAR 9/8/9, umbilical pH arterial/venous 7.32/7.34. Two of the term neonates were admitted to NICU: One patient (P3, Supplement 1) because of intrauterine hypoxia (vaginal delivery, 38 + 1 wks, 2680 g, maternal GBS detection rectal, ampicillin treatment). Another patient (P4; Supplement 1) was admitted because of acute respiratory distress syndrome/pneumonia and treated with ampicillin/gentamicin (vaginal delivery, 40 + 1 wks, 3945 g, maternal GBS detection rectal and ampicillin treatment). The maternal outcome was without complications for all 24 women.

**Table 2:** Participant characteristics (n = 23^1^) for mothers and term newborns (mean ± SD; min–max; *mean only).

	I (n = 3)	II (n = 10)	III (n = 4)	IVa (n = 3)	IVb (n = 3)
Maternal age (y)	26.3 ± 1.8; 25–29	32.2 ± 4.1; 25–39	36.3 ± 2.5; 34–40	26.3 ± 0.5; 26–27	34.0 ± 0.9; 33–35
Week of gestation	40 + 2*; 39 + 5–41 + 0	39 + 3*; 36 + 6–40 + 2	39 + 2*; 37 + 6–40 + 0	39 + 2*; 38 + 3–40 + 1	37 + 2*; 37 + 0–38 + 5
Gravida	4 ± 0.4; 3–4	2 ± 0.7; 1–3	2 ± 0.7; 1–3	3 ± 0.8; 2–4	2 ± 0.0; 2–2
Para	3 ± 1.2; 1–4	1 ± 0.7; 1–3	2 ± 0.4; 1–2	2 ± 0.9; 1–3	2 ± 0.4; 1–2
Neonatal weight (g)	3558 ± 105; 3440–3695	3527 ± 454; 2680–4165	3417 ± 284; 2973–3720	3297 ± 46; 3250–3360	3385 ± 68; 3300–3465
Neonatal length (cm)	51.6 ± 2.1; 49–54	51.9 ± 2.8; 48–56	51.5 ± 1.5; 50–54	52 ± 0.8; 51–53	51.7 ± 1.2; 50–53
Neonatal head circumference (cm)	35.0 ± 0.8; 34–36	35.2 ± 1.1; 33–37	34.8 ± 1.4; 32.5–36	34.3 ± 0.2; 34–34.5	34.8 ± 0.6; 34–35.5
APGAR 5 min	10*; 9–10	9*; 7–10	10*; 9–10	10*; 10–10	8*; 7–9
APGAR 10 min	10*; 10–10	10*; 8–10	10*; 10–10	10*; 10–10	10*; 9–10
Umbilical artery pH	7.30 ± 0.09; 7.18–7.41	7.19 ± 0.10; 6.96–7.32	7.28 ± 0.02; 7.25–7.31	7.28 ± 0.06; 7.19–7.33	7.26 ± 0.15; 7.05–7.38
Umbilical vein pH	7.38 ± 0.07; 7.31–7.48	7.36 ± 0.1; 7.22–7.51	7.34 ± 0.01; 7.32–7.35	7.36 ± 0.07; 7.25–7.42	7.38 ± 0.03; 7.33–7.40

^1^One case was excluded from statistical analysis, because of preterm birth (34 + 3 wks).

Participants from Group II (GBS positive by maternity records, vaginal delivery) were treated with antibiotics following the current German guidelines for prophylaxis of newborn GBS disease^13^. Women were treated with 2 g ampicillin i.v. (first dose) followed by 1 g ampicillin i.v. every 4 h intra partum. Number of doses given ranged from 1 to 7 (n = 10) depending on the arrival at the delivery room, rupture of membranes and time of birth.

### Microbiological results clinical approach

**Figure 1:**
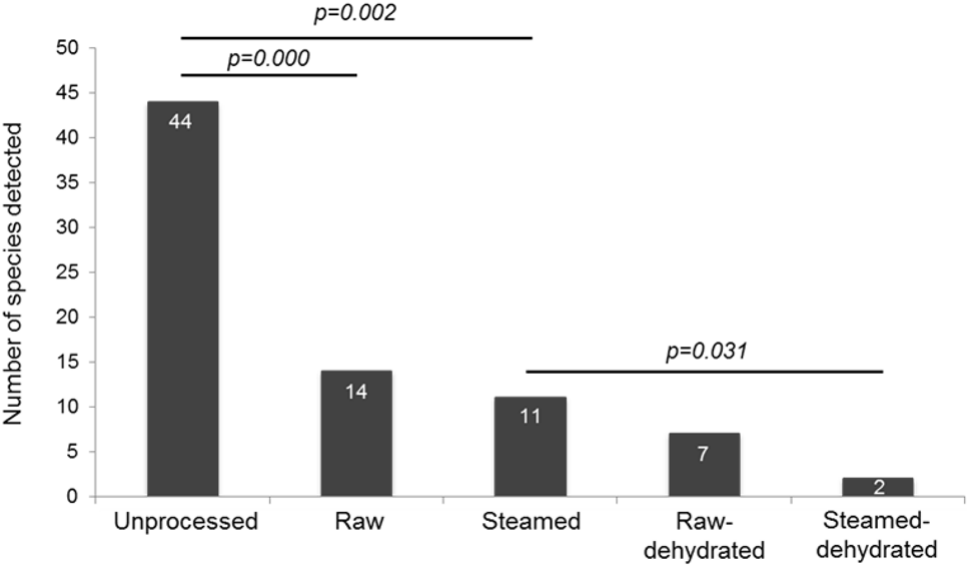
Number of species detected on unprocessed placentas (fetal and maternal side) and processed samples (n = 24) dependent on preparation methods. The tap water contaminant *Achromobacter xylosoxidans* was discounted.

Microbiological culture analysis of raw, steamed, raw-dehydrated, and steamed-dehydrated placenta tissue showed growth of various species (Fig. 1, Table 3). Unprocessed placenta harbored the highest number of microbial species (growth on fetal and maternal side). The number of microorganisms declined with further processing that includes rinsing the placenta with cold water for the preparation of raw samples (Fig. 1). The Wilcoxon test confirmed significant species reduction when unprocessed compared with raw (*p* = 0.000), with steamed (*p* = 0.002) and when steamed compared with steamed-dehydrated (*p* = 0.031) samples. Raw versus raw-dehydrated samples showed no significant reduction of microorganisms.

**Table 3:** Different species detected on raw, steamed, raw-dehydrated and steamed-dehydrated placenta preparations (n = 24).

	Delivery mode	Anti-biotic treatment	Group	Raw	Steamed	Raw-dehydrated	Steamed-dehydrated
GBS positive by maternity record	Vaginal delivery	No	I	*Kocuria kristinae, Corynebacterium* spp*., coagulase negative Staphylococci, Streptococcus anginosus, Staphylococcus epidermidis, Cryptococcus sedentarius, Pseudomonas aeruginosa*	*Staphylococcus warneri*	*Staphylococcus epidermidis*	*–*
		Yes	II	Achromobacter xylosoxidans*	Paenibacillus pabuli, Staphylococcus epidermidis, Achromobacter xylosoxidans*	*Staphylococcus epidermidis, Micrococcus luteus*	*–*
	C-Section	No	III	*Gardnerella vaginalis*	*Micrococcus luteus*	*Propionibacterium acnes*	*–*
Unscreened for GBS	Vaginal delivery	No	IVa	*Staphylococcus haemolyticus, Staphylococcus epidermidis, Cutibacterium acnes*	*Staphylococcus epidermidis, Cutibacterium acnes*	*Corynebacterium tuberculostearicum, Staphylococcus epidermidis*	*Bacillus subtilis*
	C-Section	No	IVb	*Staphylococcus lugdunensis, Staphylococcus epidermidis*	*Bacillus subtilis, Staphylococcus pasteuri, Staphylococcus epidermidis, Paenibacillus lautus*	*Cutibacterium acnes*	*Paenibacillus provencensis*

*Achromobacter xylosoxidans indicates contamination from tap water and growth was shown in surface disinfectant.

## Results experimental approach

### Placenta surface model

The raw and homogenized, non-surface-inoculated tissue showed no growth of microorganisms after 48 h; neither the cultures of the maternal and fetal side of the three placentas. One placenta (sample 1) showed meconium stain but no growth of microorganisms on the surface or in the homogenized raw tissue. Growth in colony forming units (CFU) on raw tissue after surface inoculation was shown for GBS (Table 4), as well as for *E. coli* (Table 5); raw-dehydrated tissue also showed growth for both species but in lower concentrations. None of these organisms were found on steamed or steamed-dehydrated tissue.

**Table 4:** Growth of GBS in colony forming units (CFU) from different swabs and on placenta tissue preparations.

	Sample no	Fetal	Maternal	Raw	GBS in CFU					
					Suspension	Fetal/maternal	Raw	Raw-dehydrated	Steamed	Steamed-dehydrated
Surface	1	x	x	x	**90**	**1**	x	x^1^	x	x^2^
	2	x	x	x	**90**	**12**	**35**	After enrichment	x	x
	3	x	x	x	**53**	**124**	**48**	**2**	x	x
Injection	4	**2**	x	x	**53**	n.a.	**102**	**4**	x	x^3^
	5	x	x	x	1000*	n.a.	**160**	**21**	x	x
	6	x	x^4^	x	1000*	n.a.	**43**	**13**	x	x

Further microorganisms were detected: ^1^*Bacillus* spp. 1 CFU, ^2^*Staphylococcus epidermidis* 1 CFU, ^3^*Bacillus* spp. after enrichment, ^4^*Staphylococcus haemolyticus* CFU 6. (x) no growth of GBS after 48 h; (*) bacteria per ml; (n.a.) not analyzed.

Significant values are in bold.

**Table 5:** Growth of *E. coli *in colony forming units (CFU) on different swabs and placenta tissue preparations.

	Sample no	Fetal	Maternal	Raw	*E. coli* in CFU					
					Suspension	Fetal/maternal	Raw	Raw-dehydrated	Steamed	Steamed-dehydrated
Surface	1	x	x	x	**70**	**50**	**5**	**1**	x	x
	2	x	x	x	**110**	**55**	**5**	**2**	x	x
	3	x	x	x	**13**	**14**	**4**	**1**	x	x
Injection	4	x^1^	x	x	**13**	n.a.	**82**	After enrichment	x	x
	5	x	x	x	10,000*	n.a.	**62**	**3**	x	x
	6	x	x^2^	x	10,000*	n.a.	**93**	**4**	x	x

Further microorganisms were detected: ^1^GBS 2 CFU, ^2^*Staphylococcus haemolyticus* CFU 6. (x) no growth of *E. coli* after 48 h; (*) bacteria per ml; (n.a.) not analyzed.

Significant values are in bold.

### Placenta injection model

The raw and homogenized, non-injection-impregnated tissue showed no growth of microorganisms after 48 h. Bacterial growth on raw tissue after injection-inoculation was shown for GBS (Table 4), and *E. coli* (Table 5), both in higher concentrations compared to the surface inoculation model. Again, no GBS or *E. coli* were found on steamed and steamed-dehydrated tissue.

### Influence of heat processing to GBS and *E. coli*

**Figure 2:**
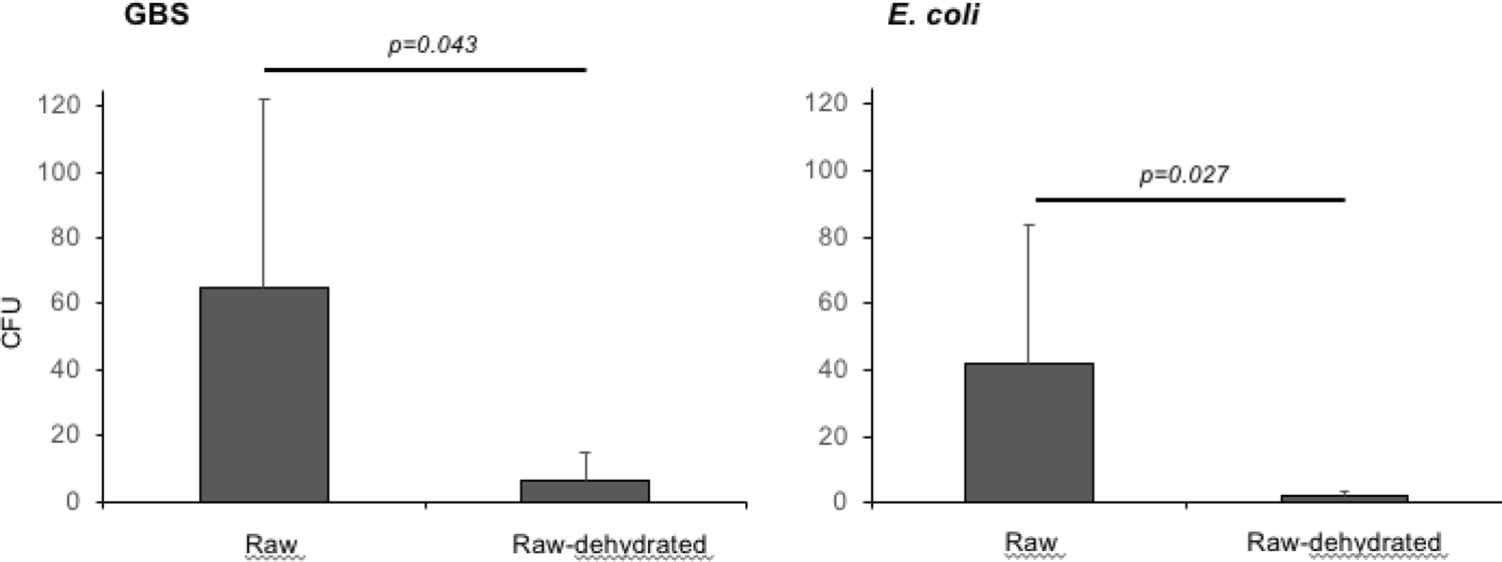
Number of CFU (mean ± SD) from raw and raw-dehydrated placenta samples after artificial inoculation (surface and injection samples merged) with GBS or *E. coli*. Raw preparation includes rinsing placenta under cold running water and homogenizing the tissue. Raw-dehydrated tissue was heated to 55 °C for 8 h.

The reduction of CFU associated with the heat processing after artificial inoculation (surface and injection inoculation merged) with GBS and *E. coli* is shown in Fig. 2. The Wilcoxon test showed significant reduction for both GBS (p = 0.043) and *E. coli* (p = 0.027) in raw compared to raw-dehydrated samples.

Steaming and steaming followed by dehydration eliminates both microorganisms completely (Tables 4 and 5).

## Discussion

In the clinical approach, placentas from GBS positive and unscreened mothers were processed to analyze bacterial tissue contamination.

As a positive GBS-status requires routinely antibiotic treatment (e.g. ampicillin i.v.), it is highly unlikely that a GBS-positive woman delivering in hospital would not be administered antibiotics. As a result, we were only able to collect three placentas to group I “GBS positive, vaginal delivery, no antibiotics”. In an effort to increase placentas from GBS positive mothers who did not receive antibiotics, we extended our placenta collection to group IV “unscreened”. However, only one out of seven cases (14%) of unscreened mothers not receiving antibiotic treatment during vaginal delivery was randomly positive for GBS. This result resembles the overall incidence of GBS-colonization (worldwide estimated 18% of women^16^).

The analysis of negative tested placentas served as an internal control group. Further, these placentas were processed to gain data from placentas born via C-section. It should be noted that placentophagy is not solely practiced by women in the homebirth community. Selander et al.^2^ report that of the 212 births (after which placentophagy was practiced by their survey participants), 72 (34%) were hospital births. In addition, Young et al. report that of the 27 total participants in their phase 1 randomized clinical trial (all of whom had decided to consume the placenta postpartum before entering the trial), five women (19%) delivered via C-section^13^.

In one case a placenta (P7, Supplement 1) from Group II (GBS positive, vaginal delivery, antibiotic treatment) showed growth of GBS on its surface. The mother received 3 times ampicillin according to the current German guidelines^18^. From this single case no prediction can be made weather a vaginal GBS transmission from the birth canal to the placenta surface occurs under antibiotic treatment. One placenta that was born via C-section without administered antibiotic treatment also showed GBS contamination. Per our internal hospital guidelines, we indeed administer antibiotics during C-section, but only after the neonate and placenta are born. While this is not consistent with the general German clinical practice, this is our hospital’s clinical routine and is not a procedure changed due to the study design.

In the two cases where placenta surfaces showed GBS growth, no contamination of raw or processed tissue occurred. Further, all processed samples were free from GBS growth independent from the maternal GBS-status.

Raw tissue showed growth of common colonizers of skin and mucosa including facultative pathogen organisms (*Gardnerella vaginalis*, *Kocuria kristinae*, *Corynebacterium* spp., coagulase negative Staphylococci, *Streptococcus anginosus, Staphylococcus haemolyticus, Staphylococcus epidermidis, Staphylococcus lugdunensis, Cutibacterium acnes, Pseudomonas aeruginosa, Achromobacter xylosoxidans*). Raw tissue from women without antibiotic treatment showed a higher diversity of microorganisms compared to women who underwent antibiotic treatment.

Raw-dehydrated tissue showed growth of *Staphylococcus epidermidis*, *Propionibacterium acnes, Micrococcus luteus, Corynebacterium tuberculostearicum, Cutibacterium acnes*—likewise colonizers of skin and mucosa and probably processing contamination. Steamed-dehydrated tissue showed growth of *Bacillus subtilis* and *Paenibacillus provencensis*.

All four tissue processing protocols significantly reduced the number of species detected on placenta samples; steaming followed by dehydration had the greatest impact on the reduction of species, which is consistent with our earlier study^9^. But even raw tissue showed significantly less microbiota compared to the number of species detected on placenta surface right after birth. This could be due to the first step of processing during the clinical study in which placentas were rinsed under cold running water with blood and blood clots removed.

Some of the raw and steamed samples showed contamination with *Achromobacter xylosoxidans*, a potential pathogenic bacteria in a wet environment. A consultation by the Institute for Clinical Hygiene was made. Growth of *Achromobacter xylosoxidans* in the tab-water and container for surface disinfectant (0.5% Mikrobac^®^ forte, Bode Chemie GmbH, Hamburg, Germany) was shown. Therefore, from placenta seven on, the disinfectant wipes were changed to Incidin™ OxyWipe S (Ecolab, Monheim am Rhein, Germany) and no further detection of this bacteria occurred.

In the series inoculation experiments, we were able to show that GBS and *E. coli* are remarkably sensitive to heat processing. The introduced model for artificial contamination of placenta tissue through injection with GBS and *E. coli* suspension lead to massive bacterial growth on raw tissue. Raw tissue from surface inoculation placentas also showed bacterial growth but in minor concentrations compared to injection inoculation. Both dehydration, and steaming followed by dehydration, significantly reduced the number of GBS and *E. coli*: steamed and steamed-dehydrated tissue of any inoculated samples showed no growth of these contaminants.

One placenta showed meconium stain, although the swabs from the fetal and maternal side showed no bacterial growth. This finding supports the notion that meconium is not infectious per se, but can be interpreted as a sign for intrauterine stress and hypoxia.

The inconclusive result in sample 4 prepared for the experimental approach for *E. coli* contamination showing GBS colonization (Table 5) has to be mentioned. From the experimental setting used it can not be excluded that GBS contamination was caused accidentally, since samples were prepared side by side with sample 3 that day. A specific PCR was not applied to the inoculated samples, as the tissue was exposed to high concentration of GBS specific DNA.

The *British Food Standard Agency* (FSA) established the consumption of placenta and placenta products by Article 2 Regulation (EC) No. 178/2002^34^ as a food^35^. In terms of food safety, however, when comparing raw placental tissue to the food category “minced meat”, where a concentration of 500 CFU/g of *E. coli* is the maximum allowed by law^36^, even raw human placenta which has only been rinsed with cold water exhibit *E. coli* concentrations that are a small fraction of this limit. Steamed and steamed-dehydrated human placenta showed no detectable colonization of *E. coli*.

Although evidence for GBS as a foodborne pathogen exist, GBS are remarkably sensitive to heat preparation. Compared to *E. coli*, GBS strains are much more susceptible to mild heat treatment (56 °C) and acid stress. Therefore, the risk of GBS as a foodborne pathogen can be addressed through modest heating temperatures^32,33^. Further, GBS are characterized through low acid resistance in gastric milieu with pH of 2.35 of gastric fluid^29^ that has to be distinguished from GBS existence in other low pH environments such as the flora of the recto-vaginal mucosa (pH 3.5–4.5). To date, the infectious dose for GBS as a foodborne pathogen in humans is undetermined. The poor survivability of GBS under acidic stress has led to the hypothesis that a relatively high infectious dose may be required to cause foodborne disease^32^.

While transfer of streptococci from maternal gut to breast milk has been shown^37^ only massive numbers of GBS transmitted to the infant can cause GBS disease^38^. The rare transmission of GBS from the mother to the newborn via breast milk can occur through high maternal colonization during maternal GBS disease, most often acute endometritis, but also mastitis^38,39^. While GBS transmission through close contact with colonized persons can not ruled out, the intake of minimal amounts of placental tissue (approximately 3 g of pulverized tissue daily^11^) with a low GBS load as cause for maternal bacteremia disease seems improbable. This is consistent with the analysis of 23.525 data sets from the United States midwives’ medical records where placentophagy was not associated with any negative neonatal outcomes^3^.

The analysis of steamed samples was included to investigate the effect of high thermal processing to the tissue, though it has minor importance for practical use. A historical Chinese medical encyclopedia describes the use of dehydrated placenta *humanum* in combination with herbal drugs^40^, but the process of steaming followed by dehydration seems a modern interpretation of the documented historic preparations.

Simple processing, such as rinsing with cold water, and to a much greater extent, heat processing, significantly reduces overall microbial species and growth of GBS and *E. coli* on placenta tissue thus lowering the risk of microbial contamination. This finding is consistent with the use of traditional thermal processes like pasteurization (63 °C for 30 min or 72 °C for 15 s) that are used to minimize microorganisms and are known to destroy pathogenic bacteria like *E. coli* and Streptococcus species^41,42^. Our previous study also showed that the opportunistic potentially pathogenic yeast *Candida albicans* was eliminated through heat preparation^9^. These results do not support placentophagy of processed tissue as a likely source of clinical infection. If placentophagy is requested, the preparation processes, especially heat processing, appear to minimize potentially pathogen microorganisms even through lower temperature dehydration of ’raw’ placental tissue at 55 °C for 8 h. While placenta tissue is not sterile, the maternal exposure to requisite amounts of pathogens to cause disease is improbable.

In conclusion, although our experimental study is limited by the low number of processed placentas and participant recruitment due to German clinical regulations (which do not accurately reflect the real-life placentophagy community), our results confirm that placentophagy of heat-processed tissue is an unlikely source of clinical infection.

## Materials and methods

### Clinical approach

#### Placenta donors and inclusion criteria

This study was approved by the ethics committee of the University of Jena (ethical approval no. 2018-1069) and informed consent has been obtained from each placenta-donor. All methods were performed in accordance to the Declaration of Helsinki. 24 placentas from singleton pregnancies were collected and processed from April 2018 until June 2020. To verify the GBS-status documented in the expectant mothers record of prenatal and natal care (“Mutterpass”), mothers were screened voluntarily again for GBS by vaginal and rectal swab at time of birth in the delivery room. Participants were divided in four groups:

(I) GBS positive, vaginal delivery, no antibiotic treatment
(II) GBS positive, vaginal delivery, antibiotic treatment
(III) GBS positive, C-section, no antibiotic treatment
(IV) unscreened for GBS, vaginal delivery, no antibiotic treatment
(V) unscreened for GBS, C-section, no antibiotic treatment.

#### Sample collection and preparation

To resemble the most common method of placenta preparation in a home-based environment, the processing was done under clean but not sterile conditions. Placentas were taken immediately after birth to the placenta lab in clean, disinfected containers. Within 2 h an eSwab containing Amies transport medium (Copan, Brescia, Italy) was used to take a smear from the maternal and fetal side of the placenta. Subsequently, the placenta was washed under cold running tap water and blood and blood clots were removed. The placenta was cut into three pieces for the preparation of raw, raw-dehydrated, steamed and steamed-dehydrated samples; the umbilical cord and amnion were excised. To evaluate the impact of steaming, a piece of tissue was excised from the steamed placenta third that was further dehydrated for the analysis of steamed-dehydrated tissue. The processing was done in accordance with previously published methods^9^, except the dehydration duration was reduced to 8 h.

#### Microbiological analysis

Samples from raw, steamed, raw-dehydrated and steamed-dehydrated tissue, eSwab samples collected from the maternal and fetal side of the placenta and vaginal and rectal eSwab samples taken from the mother were analyzed by standard routine culture procedures to identify potentially pathogenic bacteria and fungi. The placenta tissue and swab samples were streaked onto Columbia sheep blood agar, chocolate agar, Drigalski lactose agar, Schaedler agar, (Oxoid, Thermo Fisher Scientific) Enrichment culture was used to detect bacteria at low concentrations. Brain–heart infusion broth (BHI; BD, Heidelberg, Germany) was inoculated with the powder from steamed, dehydrated and ground placental tissue. Blood and chocolate agar plates were incubated at 37 °C at aerobic conditions with 5% CO_2_ for 48 h. Drigalski agar plates were incubated at aerobic conditions for 24 h. Cultures on Schaedler agar were incubated at anaerobic conditions for 96 h. BHI broth was streaked onto blood and chocolate agar after overnight incubation.

These culture media are appropriate to isolate common microbial species of the normal vaginal flora as well as *Staphylococcus aureus*, hemolytic streptococci, enterococci, Enterobacteriaceae including enteropathogenic species, non-fermenters including *P. aeruginosa, Candida* spp. and filamentous fungi. An additional culture media was used for culturing Candida species: CHROMagar Candida Plus (Mast Diagnostica, Reinfeld, Germany). Identification of bacteria and fungi was performed using the examination of specific colony morphologies, characteristic growth on differential and selective media, and further species identification with Matrix Assisted Laser Desorption Ionization Time-of-Flight (MALDI-TOF) mass spectrometry (Vitek MS, bioMerieux, Nürtingen, Germany) if relevant^43^. A classical enumeration technique by manual colony counting was also used.

Antimicrobial Susceptibility Testing was performed using Vitek 2 and minimal inhibitory concentration interpretation according to European Committee on Antimicrobial Susceptibility Testing (EUCAST criteria)^44^.

#### Molecular method (Alethia^®^-Test)

To identify GBS in the samples molecular diagnostics was additionally performed using the FDA-approved Alethia^®^ Group B Streptococcus assay based on isothermal loop-mediated amplification (LAMP) (Meridian Bioscience, distributed by Virotech, Rüsselsheim, Germany). Samples were frozen at − 80 °C until the collection phase was completed and an adequate amount of samples were taken. A volume of 50 µl of eSwab transport medium or BHI broth inoculated with tissue samples was added to 200 µl of Alethia^®^ control reagent and incubated at 95 °C for 10 min. After vortexing 50 µl of the suspension was transferred into the reaction buffer. Each 50 μl of the reaction mix was pipetted into both the test and internal control chamber of the test device containing lyophilized master mixes. The test was run in an illumipro-10 machine (Meridian Bioscience) for 40 min.

### Experimental approach

For artificial inoculation six placentas born via C-section were used and swabbed right after birth, four from singleton pregnancies, two from twin pregnancy. Standardized saline-suspensions (0.9%) of highly concentrated (total 10^5^ CFU each) GBS and *E. coli* bacteria were prepared by the Institute for Infectious Diseases and Infection Control of the Jena University Hospital. The solution was prepared after standard dilution procedures: Bacteria were incubated on a blood agar plate at 37 °C over night. On the next day, 4–5 colonies were inoculated into 10 ml MH (Müller Hinton) media and incubated under shaking conditions at 37 °C and 160 rpm for 3–4 h. Then optical density (OD600) was determined and bacterial suspension was adjusted to 10^5^ CFU/ml. The exact concentration of bacteria in the solution was proven by plating.

The choice of strains resulted from the following clinical parameters: GBS and *E. coli* were isolated from blood-cultures of adult patients diagnosed with sepsis. The experiments described below were done under clean but not sterile conditions.

**Figure 3:**
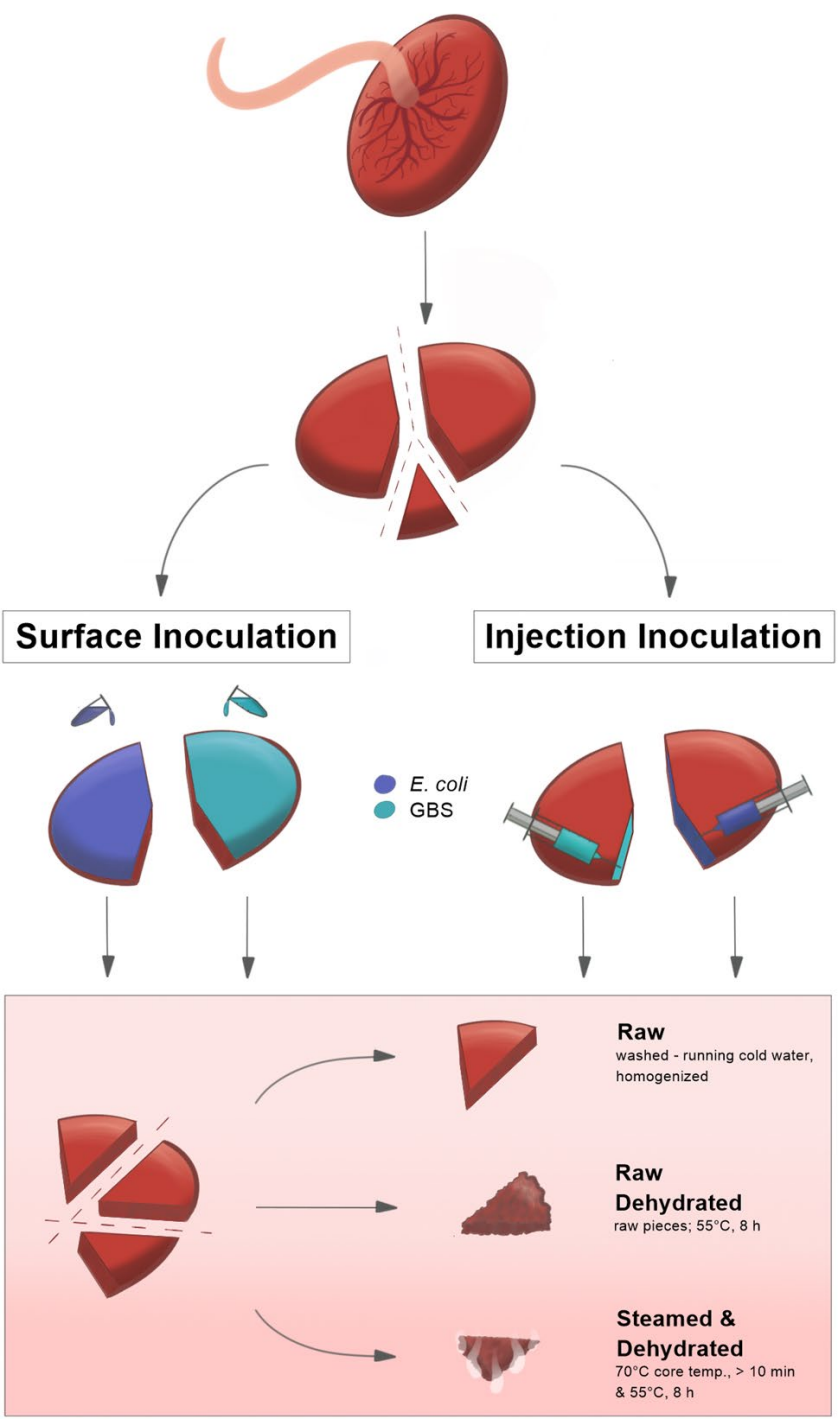
Standardized model for artificial placenta inoculation. *Surface inoculation*: 5 ml of GBS or *E. coli* suspension (total 10^5^ CFU each) were manually rubbed onto placenta surface (maternal and fetal side), then the placenta left covered for 30 min. Control eSwabs from the maternal and fetal side of the inoculated placenta were taken. *Injection inoculation*: 5 ml of GBS or *E. coli* suspension (total 10^5^ CFU each) were injected into the intervillous space: 1 ml was injected at five evenly spread places of the placenta piece. As the placenta was inoculated through injection, no surface swabs were taken. Note: unlike the placentas obtained in the clinical arm of the study, inoculated placentas were not rinsed under cold running water. The inoculated pieces were then further processed as described in detail in our earlier publication^5^: one third was homogenized for analysis of raw tissue. Another third was dehydrated for 8 h at 55 °C. The last third was steamed for at least 10 min until the core temperature was above 70 °C, a small piece for analysis excised and afterwards dehydrated for 8 h at 55 °C.

To resemble possible hypotheses of placental GBS colonization, two inoculation methods were utilized: Inoculation by placenta surface (according to vaginal ascending colonization hypothesis) and inoculation by placenta injection (according to blood stream colonization hypothesis). For each method n = 3 placentas were inoculated and processed as described in Fig. 3.

### Inoculation method by placenta surface model and placenta injection model

Immediately after placenta birth eSwabs were taken from the maternal and fetal side of the placenta. Then one piece was excised for microbial analysis without impregnation. The placenta was divided and inoculated with 5 ml of GBS or *E. coli* suspension (total 10^5^ each) through two different methods as shown in Fig. 3. (Note: unlike the clinical approach of the study, inoculated placentas were not washed.) Placentas were processed following the protocol detailed in a previously published study^9^, dehydration duration was reduced to 8 h.

### Statistical analysis

The Wilcoxon signed-rank test was used to validate the reduction of microorganisms on placenta tissue between two different preparation methods from one placenta.

## Supplementary Information

Supplementary Information.

## Abbreviations

APGAR: Backronym for “Appearance, Pulse, Grimace, Activity, Respiration”
CDC: Centers for Disease Control and Prevention
CFU: Colony forming units
*E. coli*: *Escherichia coli*
EOD: Early onset disease
FSA: Food Standard Agency
GBS: Group B streptococci
i.v.: Intravenous
LAMP: Loop-mediated amplification
LOD: Late onset disease
MALDI-TOF: Matrix Assisted Laser Desorption Ionization Time-of-Flight
PROM: Premature rupture of membranes
wks: Weeks of gestation
