# Supplementary material for: Impact of tissue processing on microbiological colonization in the context of placentophagy
Source: Sci Rep. 2022 Mar 29;12:5307. doi: 10.1038/s41598-022-09243-4 (PMC8964728; doi:10.1038/s41598-022-09243-4)
Supplement: Supplementary file 1 — Supplementary Information. [file 41598_2022_9243_MOESM1_ESM.docx]

**Supplement 1: GBS results for all placentas and preparations analyzed.** GBS-detection in placenta samples were done with two different methods (A) conventional culture technique and (B) isothermal loop-mediated amplification (LAMP).

P5 was excluded and not further analyzed because of the negative maternal GBS- status. P3: NICU admission of infant 38+1 wks with intrauterine hypoxia; vaginal delivery, 2680 g, maternal GBS detection rectal, ampicillin treatment and P4: NICU admission of infant 40+1 wks with acute respiratory distress syndrome/pneumonia, treated with ampicillin/gentamicin, vaginal delivery, 3945 g, maternal GBS detection rectal, ampicillin treatment. P18: Preterm infant 34+3 wks born via C-section, mother unscreened for GBS, no GBS detection vaginal/rectal, Gravida/Para status II/I, weight 1835 g, length 42 cm, head circumference 29 cm, APGAR 9/8/9, umbilical pH arterial/venous 7.32/7.34. (*) invalid, (**) not tested

|  | **Group** |  | **vaginal** | **rectal** | **fetal** | **maternal** | **raw** | **raw- dehydrated** | **steamed** | **steamed- dehydrated** |
| --- | --- | --- | --- | --- | --- | --- | --- | --- | --- | --- |
| **P1** | **II** | A | - | + | - | - | - | - | - | - |
|  |  | B | - | + | - | - | - | - | - | - |
| **P2** | **III** | A | - | - | - | - | - | - | - | - |
|  |  | B | - | - | - | - | - | - | - | - |
| **P3** | **II** | A |  | + |  |  |  |  |  |  |
|  |  | B | - | - | - | - | * | * | - | - |
| **P4** | **II** | A | - | + | - | - | - | - | - | - |
|  |  | B | - | + | - | - | - | - | - | - |
| **P5** | excluded |  |  |  |  |  |  |  |  |  |
| **P6** | **II** | A | + | - | - | - | - | - | - | - |
|  |  | B | - | + | - | - | - | - | - | - |
| **P7** | **II** | A | - | - | - | + | - | - | - | - |
|  |  | B | + | + | - | - | - | - | - | - |
| **P8** | **II** | A | - | - | - | - | - | - | - | - |
|  |  | B | - | - | - | - | - | - | - | - |
| **P9** | **III** | A | + | + | + | - | - | - | - | - |
|  |  | B | + | + | - | - | - | - | - | - |
| **P10** | **I** | A | - | - | - | - | - | - | - | - |
|  |  | B | - | - | - | - | - | - | - | - |
| **P11** | **IVa** | A | - | - | - | - | - | - | - | - |
|  |  | B | - | - | - | - | - | - | - | - |
| **P12** | **I** | A | - | - | - | - | - | - | - | - |
|  |  | B | - | - | - | - | - | - | - | - |
| **P13** | **IVb** | A | - | - | - | - | - | - | - | - |
|  |  | B | - | - | - | - | - | - | - | - |
| **P14** | **IVb** | A | - | - | - | - | - | - | - | - |
|  |  | B | - | - | - | - | - | - | - | - |
| **P15** | **IVb** | A | - | - | - | - | - | - | - | - |
|  |  | B | - | + | - | - | - | - | - | - |
| **P16** | **II** | A | + | - | - | - | - | - | - | - |
|  |  | B | + | + | - | - | - | - | - | - |
| **P17** | **IVa** | A | - | - | - | - | - | - | - | - |
|  |  | B | - | - | - | - | - | - | - | - |
| **P18** | **IVb** | A | - | - | - | - | - | - | - | - |
|  |  | B | - | - | - | - | - | - | - | - |
| **P19** | **III** | A | + | ** | - | - | - | - | - | - |
|  |  | B | + | ** | - | - | - | - | - | - |
| **P20** | **III** | A | + | - | - | - | - | - | - | - |
|  |  | B | + | + | - | - | - | * | - | - |
| **P21** | **I** | A | - | + | - | - | - | - | - | - |
|  |  | B | - | - | - | - | - | * | * | * |
| **P22** | **IVa** | A | - | - | - | - | - | - | - | - |
|  |  | B | - | - | - | - | - | - | - | - |
| **P23** | **II** | A | - | - | - | - | - | - | - | - |
|  |  | B | - | - | - | - | - | * | - | - |
| **P24** | **II** | A | - | + | - | - | - | - | - | - |
|  |  | B | + | - | - | - | - | * | - | - |
| **P25** | **II** | A | + | + | - | - | - | - | - | - |
|  |  | B | + | + | - | - | - | * | - | - |
|  |  |  |  |  |  |  |  |  |  |  |
|  |  |  |  |  |  |  |  |  |  |  |
